# Hypertriglyceridemia: Molecular and Genetic Landscapes

**DOI:** 10.3390/ijms25126364

**Published:** 2024-06-08

**Authors:** Pietro Scicchitano, Francesca Amati, Marco Matteo Ciccone, Flavio D’Ascenzi, Egidio Imbalzano, Riccardo Liga, Stefania Paolillo, Maria Concetta Pastore, Andrea Rinaldi, Anna Vittoria Mattioli, Matteo Cameli

**Affiliations:** 1 Cardiology Department, Hospital “F Perinei” ASL BA, 70022 Altamura, Italy; 2 Cardiovascular Diseases Section, University of Bari, 70124 Bari, Italy; francesca.amati86@gmail.com (F.A.); marcomatteo.ciccone@uniba.it (M.M.C.); 3 Department of Medical Biotechnologies, Division of Cardiology, University of Siena, 53100 Siena, Italy; flavio.dascenzi@gmail.com (F.D.); pastore2411@gmail.com (M.C.P.); matteo.cameli@unisi.it (M.C.); 4 Department of Clinical and Experimental Medicine, University of Messina, 98122 Messina, Italy; eimbalzano@unime.it; 5 Department of Surgical, Medical, Molecular Pathology and Critical Area, University of Pisa, 56126 Pisa, Italy; riccardo.liga.dott@gmail.com; 6 Department of Advanced Biomedical Sciences, University of Naples “Federico II”, 80138 Naples, Italy; paolilloste@gmail.com; 7 Unit of Cardiology, Department of Experimental, Diagnostic and Specialty Medicine-DIMES, University of Bologna, Sant’Orsola-Malpighi Hospital, IRCCS, 40138 Bologna, Italy; rinaldiandrea19@gmail.com; 8 Department of Science of Quality of Life, University of Bologna “Alma Mater Studiorum”, 40126 Bologna, Italy; annavittoria.mattioli@unibo.it

**Keywords:** molecular mechanisms, genetics, physiopathology, triglycerides, cardiovascular diseases

## Abstract

Lipid disorders represent one of the most worrisome cardiovascular risk factors. The focus on the impact of lipids on cardiac and vascular health usually concerns low-density lipoprotein cholesterol, while the role of triglycerides (TGs) is given poor attention. The literature provides data on the impact of higher plasma concentrations in TGs on the cardiovascular system and, therefore, on the outcomes and comorbidities of patients. The risk for coronary heart diseases varies from 57 to 76% in patients with hypertriglyceridemia. Specifically, the higher the plasma concentrations in TGs, the higher the incidence and prevalence of death, myocardial infarction, and stroke. Nevertheless, the metabolism of TGs and the exact physiopathologic mechanisms which try to explain the relationship between TGs and cardiovascular outcomes are not completely understood. The aims of this narrative review were as follows: to provide a comprehensive evaluation of the metabolism of triglycerides and a possible suggestion for understanding the targets for counteracting hypertriglyceridemia; to describe the inner physiopathological background for the relationship between vascular and cardiac damages derived from higher plasma concentrations in TGs; and to outline the need for promoting further insights in therapies for reducing TGs plasma levels.

## 1. Introduction

Triglycerides (TGs) are lipid compounds basically used by organs as an energy depot, while acting as a protecting barrier for preserving organs’ integrity from mechanical pain [1]. Their sources are both endogenous and exogenous: the liver is the main organ involved in the synthesis of free fatty acids (FFA), which are the fundamental constituents for TGs; food is the external delivery resource for TGs, as TGs included in foods are absorbed into the bowel, and move on into the body by means of lipoproteins and the lymphatic system [1].

Alterations in the metabolism of TGs are the result of several conditions, including abnormalities in food intake or, rather, genetic conditions that impair the complex, biochemical pathways which regulate the synthesis, processing, and degradation of TGs [1].

The increase in TG serum levels can be as dangerous as conditions involving cholesterol [2,3,4,5,6,7]. The risk for coronary heart diseases (CHD) can range from 57 to 76%, according to the literature data [8]. Specifically, the data demonstrated that the higher the plasma concentrations in TGs, the higher the prevalence and incidence rate of major adverse cardiac events such as death, myocardial infarction, and stroke [9,10].

Specifically, the residual cardiovascular risk related to higher plasma levels in TGs is higher, independent of low-density lipoprotein-cholesterol (LDL-C) levels. The Progression of Early Subclinical Atherosclerosis [PESA] study found that TG levels ≥150 mg/dL are related to a 35% increase in subclinical noncoronary atherosclerosis risk [11]. Indeed, even at TG plasma levels lower than 150 mg/dL, there is a significant increase in cardiovascular risk [12,13]. Therefore, the landscape of atherosclerosis is currently changing, with a significant increase in patients with a lipid profile characterized by hypertriglyceridemia with a low LDL-C value.

Nevertheless, while cholesterol metabolism prevails in the general context of cardiovascular prevention, TG plasma levels often play a marginal role [14].

TGs often appear as the Cinderella of the overall panorama of lipids, thus neglecting their fundamental role in cardiac and vascular diseases which they demonstrate when higher concentrations are in the bloodstream.

The comprehension of the metabolism of TGs and the physiopathologic features underneath cardiac and vascular damages related to their higher plasma concentrations are still under investigation.

The aims of this narrative review were as follows: to provide a comprehensive evaluation of the metabolism of triglycerides and a possible suggestion for understanding the targets for counteracting hypertriglyceridemia (HTG); to describe the inner physiopathological background for the relationship between vascular and cardiac damages derived from higher plasma concentrations in TGs; and to outline the need for promoting further insights in therapies for reducing TGs plasma levels.

## 2. Methods

The database consulted was MEDLINE (https://www.nlm.nih.gov/medline/medline_overview.html (accessed on 3 June 2024)). The main key words adopted were as follows: triglycerides, hypertriglyceridemia, endothelial function, cardiomyocytes, genetics, metabolism, cardiovascular diseases. All terms were combined with each other in order to obtain the maximum selection about this argument. One physician analyzed all the studies from MEDLINE, combining each term in order to reach a complete overview on the literature background. 

## 3. Triglycerides: From Biochemical Structure to Metabolic Pathways

TGs are defined as esters deriving from the combination of glycerol with three fatty acids. Their synthesis is the combination of glucose and lipid degradations: the glucose is the main compound transformed until the creation of TGs. Two metabolic pathways are involved in the generation of diacylglycerol, i.e., the precursor of TGs: the monoacylglycerol- and the glycerol-phosphate pathways [1]. The monoacylglycerol pathway directly produced diacylglycerol (DAG) through covalent bonding monoacylglycerol and fatty acyl-CoA through the acyl-coA:monoacylglycerol acyltransferase (MGAT) [15,16,17]. Three isoforms of MGAT (MGAT1, MGAT2, and MGAT3) are known [17], which are differently expressed in adipose tissue, stomach, kidney, liver—MGAT1—and small intestine—MGAT2 and MGAT3; despite the marginal role in TGs synthesis as compared to the glycerol-phosphate pathway, the role of MGAT is fundamental in order to promote the re-conversion of fatty acids assumed with diet into TGs to be included in chylomicrons [17]. Blocking MGAT would probably mitigate diet-induced obesity and glucose intolerance, although only preclinical evidence can be outlined and human research is still lacking [18]. Glycerol-phosphate is the further biochemical pathway involved in DAG synthesis and, consequentially, TGs generation. The glycerol-3-phosphate is transformed into DAG by means of glycerolphosphate acyltransferase (GPAT) and acylglycerol-phosphate acyltransferase (AGPAT), with each enzyme responsible for adding one fatty acid CoA. The final product, i.e., the phosphatidate, is then converted into DAG by means of phosphatidic acid phosphohydrolase-1 (PPH-1).

The two biochemical pathways convey into DAG. The final step is the conversion of DAG in TG by means of diacylglycerol acyltransferase (DGAT) and acyl-CoA. Two isoforms are for DGAT: DGAT1 and DGAT2 [19]. Their homology in sequence is about 35%: DGAT1 is mostly expressed in the small intestine and adipose tissue, but less in the liver; the opposite is true for DGAT2, which is highly expressed in hepatocytes and adipocytes [19]. The amount in monoacylglycerol (MAG) and the reciprocal amount in TG/MAG may influence the activity of DGAT1 and DGAT2 [20]. Indeed, the behavior of DGAT1 and DGAT2 seems different: DGAT1 is mainly inhibited by higher concentrations in MAG and, therefore, DAG; on the opposite side, DGAT2 constitutively increases DAG production from MAG [20].

**Figure 1:**
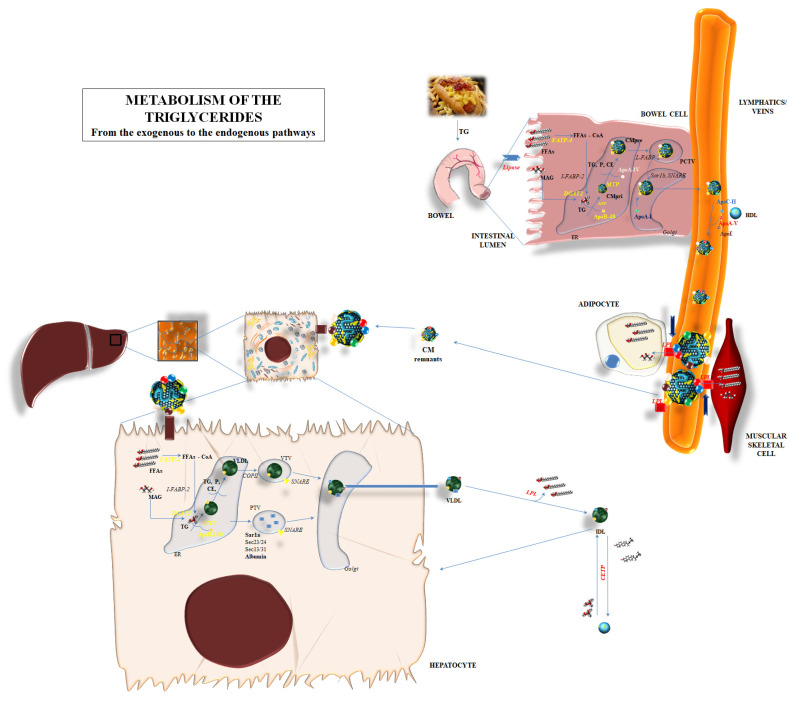
Metabolism of the triglycerides: the endogenous and exogenous pathways are represented, depicting the main biochemical mechanisms involved in the degradation, synthesis, and passage through organs of triglycerides. Abbreviations: ApoA-I: apolipoprotein A-I; ApoA-IV: apolipoprotein A-IV; ApoA-V: apolipoprotein A-V; ApoB-48: apolipoprotein B-48; ApoB-100: apolipoprotein B-100; ApoC-II: apolipoprotein C-II; ApoE: apolipoprotein E; CE: cholesteryl ester; CETP: cholesteryl ester transfer protein; CM: chylomicron; CMpri: primordial chylomicron; CMpre: pre-chylomicron; COPII: coat protein complex-II; DGAT2: diacylglycerol acyltransferase 2; ER: endoplasmic reticulum; FATP-4: fatty acid-transport protein 4; FFAs: free fatty acids; FFAs-CoA: free fatty acids–Coenzyme A; HDL: high-density lipoprotein; IDL: intermediate-density lipoprotein; I-FABP-2: intestinal fatty acid-binding protein plasma membrane-2; LPL: lipoprotein lipase; MAG: monoacylglycerol; MTP: microsomal triglyceride transfer protein; P: phospholipid; PCTV: pre-chylomicron transport vesicle; PTV: protein transport vesicles; SNARE: soluble N-ethylmaleimide-sensitive factor attachment protein receptor; TG: triglycerides; VLDL: very low-density lipoprotein; VTV: VLDL transport vesicles.

The synthesis of TGs is just the premix for the correct comprehension of the entire process related to TGs metabolism (Figure 1).

The metabolic pathway of TGs is a circle involving the synthesis, degradation, and re-synthesis of TG, balancing the exogenous and endogenous pathways for TG supply to the human body (Figure 1).

Briefly, diet supplies the most important source of TGs. Nevertheless, TGs cannot be directly absorbed via intestinal cells but rather they should be first shared into FFAs and glycerol via dedicated lipases and soon after be absorbed through intestinal cells. FFAs and glycerol can enter into the cytoplasm of the enterocytes. The transcytosis might involve a different mechanism. Passive diffusion is mainly considered as the most effective transport mechanism for FFA and MAG into enterocytes and it seems regulated by the concentration gradient [21]. Indeed, some proteins can facilitate the passage: fatty acid-binding protein plasma membrane (FABPpm), fatty acid-transport protein 4 (FATP4), fatty acid translocase/cluster determinant 36 (FAT/CD36), and/or caveolins, which are able to interact with CD36 [21]. All of these mechanisms are independently responsible for the admittance of FFA and MAG into enterocytes: the lack of one of them does not influence the transport. 

After the entrance, long-chain fatty acid transport protein 4 (FATP4)—a membrane protein also called very long-chain fatty acyl-CoA synthetase 5—esterifies long and very-long-chain fatty acids with Coenzyme A (FFA-CoA). Then, intestine-specific FABP-2 (I-FABP-2) and similar proteins are responsible for the inclusion of FFA-CoA into the endoplasmic reticulum (ER) in order to promote the synthesis of the “chylomicrons”, i.e., the lipoproteins used for the systemic widespread distribution of dietary lipids. MGAT2/3 and DGAT1 are the enzymes mainly involved in the conversion of FFAs and MAG in DAG and TG, respectively [21].

The synthesis of chylomicrons is the further step in the absorption process of lipids, and TG in particular, from the bowel. ApoB-48, encoded by the APOB gene but post-transcriptionally truncated in length (about 48%) compared to the liver-produced ApoB-100, is assembled into ER and starts the creation of chylomicron [21]. The assembly process is mediated by the microsomal triglyceride transfer protein (MTTP) [22]; derived from the MTTP gene, it is formed by two subunits: one formed by 894 amino acids and mainly expressed into liver and bowel cells, and the second, named protein disulfide isomerase (PDI), which confers stability and solubility to the final products [22]. MTTP transfers triglycerides, cholesteryl ester (CE), and phospholipids to the primordial chylomicron—or to very low-density lipoprotein (VLDL) in its liver localization—and, in parallel, includes ApoB-48 (ApoB-100 in the liver). Enterocytes also promote the inclusion of a 46-kDa protein named apoA-IV: the role of apoA-IV is still under investigation, but research has outlined the influence of such a protein in including and packaging lipids into the growing chylomicrons, making these particles larger and stable [23]. The pre-chylomicron composed of TG, CE, phospholipids, apoB-48, and apoA-IV needs to upgrade to reach its definite form; a pre-chylomicron transport vesicle leads the pre-chylomicron to the cis-Golgi apparatus by means of L-FABP. Here, the final step is the inclusion of apoA-I to the lipoprotein; the mature chylomicron can be released to the lymph directly from the Golgi apparatus. The interaction with a high-density lipoprotein (HDL) provides them with further proteins which are fundamental for their functional performances: apoC-II and apoE in particular, then apoC-I and apoC-III. In particular, chylomicrons transfer FFA to adipose tissue and muscular cells by interacting with lipoprotein lipase (LPL) through different regulatory co-factors; apoC-II and apoA-V are able to promote the lipolytic action of LPL, while apoC-I and apoC-III seem to act as inhibitors [24,25]. LPL activation promotes the degradation of a great part of TG content, the return of apoC to HDL, and the transformation of mature chylomicrons into the smaller “remnants”. Apo-E mediated the re-uptake of remnants from the liver, with the low-density lipoprotein receptor (LDLR) and LDL receptor-related protein (LRP) probably interacting in this process. This final step promotes the release of the remaining TG into the liver cells and the transformation of them into FFAs and MAG (Figure 1).

The release of FFAs into liver cells can induce a de novo lipogenesis, i.e., the creation of new TGs to be incorporated into VLDL—thus the widespread of these lipids into systemic tissues—or their degradation by means of a β-oxidation process [26]. VLDLs are primarily synthesized into hepatocyte ER; MTTP mediated the primordial creation of VLDL by including ApoB-100 and lipids, in particular triglycerides [26]. The lipid content is fundamental for the final destination of the primordial VLDL; a reduced lipid concentration in VLDL induces this lipoprotein and ApoB-100 to soon be destroyed. Heat shock protein (hsp)-70 and -90 are deputed to the degradation of incomplete VLDL [27]. When the lipid content is right, the coat protein complex-II (COPII) on the ER membrane mediates the inclusion of nascent VLDL into VLDL transport vesicles (VTV), which transport them to the cis-Golgi. Meanwhile, protein transport vesicles (PTV) lead proteins to the cis-Golgi for post-transcriptional modifications. Therefore, the VLDL content and the action of ER proteins such as Sar1, Sec23-sec24, and Sec-13 and Sec-31 are the fundamental actors in the translating process of VLDL to the cis-Golgi [26]. Specific soluble N-ethylmaleimide-sensitive factor attachment protein receptor (SNARE) proteins are responsible for the inclusion of VTV into Golgi. After maturation into Golgi, VLDL can be reversed into the bloodstream. LPL mediates the release of FFAs to the peripheral tissue; this leads to the transformation of VLDL into an intermediate-density lipoprotein (IDL). Furthermore, the cholesteryl ester transfer protein (CETP) mediates the transfer of TG to IDL and the passage of cholesteryl ester (CE) into high-density lipoprotein (HDL). CETP exerts a crucial role in the relationship between the contents of HDL and VLDL/chylomicron; the increase in CE in chylomicron and VLDL leads to the relative increase in the levels of TG into HDL. Therefore, the amount in HDL-cholesterol (HDL-C) would be apparently reduced in the case of hypertriglyceridemia (Figure 1).

## 4. Hypertriglyceridemia: Definition, Classification, and Molecular Background

HTG is defined as the pathologic increase in TGs plasma levels. According to the European Atherosclerosis Society (EAS)/European Society of Cardiology/ESC), HTG can be classified into the following: “normal” when TGs plasma levels are <1.7 mmol/L (<150 mg/dL); “mild-to-moderate” when TGs plasma levels range between 1.7 mmol/L (150 mg/dL) and <10 mmol/L (880 mg/dL); or “severe” when TG concentration overcomes >10 mmol/L (>880 mg/dL) [28].

These values represent the cut-off to be considered for trying to counteract the negative consequences of HTG. The occurrence of end-organ damages in the case of severe forms of HTG should be taken into consideration [29]. Higher plasma values in TGs promote FA overproduction, which promotes pancreatic inflammation, tissue injury, and the final expression of the features of acute pancreatitis [29]. Furthermore, a study [30] demonstrated that elevated TGs increase the risk of hepatic steatosis and NAFLD. Finally, HTG might promote a significant increase in long-term kidney function deterioration, thus leading to an increased risk of a worsening function of the human emunctory organ [31].

**Figure 2:**
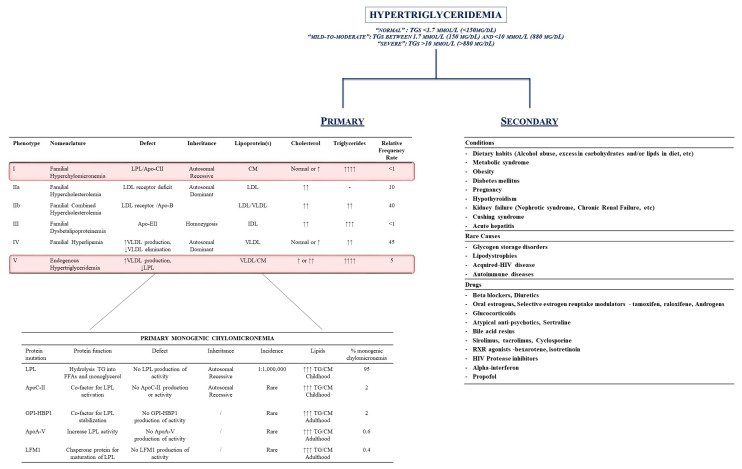
Hypertriglyceridemia (HTG): definition and classification. Hypertriglyceridemia is classified as primary or secondary to conditions whose removal can theoretically reduce the increase in triglycerides plasma levels. Inherited conditions represent the main source for primary HTG: the left side of the figure outlines the Fredrickson’s classification and the genetic background of chylomicronemias. The right side of the figure represents the secondary causes for HTG. Upward arrows indicates the increase in the characgeristics. Downward arrows indicate the decrease. Abbreviations: ApoA-V: apolipoprotein A-V; ApoC-II: apolipoprotein C-II; ApoE-II: apolipoprotein E-II; CM: chylomicron; GPI-HBP1: glycosylphosphatidylinositol-anchored high-density lipoprotein-binding protein 1; HIV: Human Immunodeficiency Virus; LFM1: Chaperone protein for maturation of LPL; LDL: low-density lipoprotein; LPL: lipoprotein lipase; RXR: retinoid X receptor; TG: triglycerides; VLDL: very low-density lipoprotein.

Therefore, HTG might be generally divided into two categories: primary (mainly genetic disorders related to alterations in genes and proteins involved in the metabolism of these lipids) or secondary conditions (i.e., diseases and/or lifestyle habits which interfere with the regulation of TGs plasma levels) (Figure 2 [28,32,33,34,35]).

Beyond primary and secondary causes of hypertriglyceridemia, the increase in TG plasma levels is a dynamic process which is based on the natural intake from foods and adaptations of tissues to foreign introduction [36,37]. Chylomicrons and VLDL production are effectively low when dealing with fasting situations, while steadily increasing in terms of number and size in the case of food intake. Specifically, foods promote the production of large-sized chylomicrons, while burst-in-remnant chylomicrons in the liver promote the synthesis of high size VLDL1 [36]. The transient and dynamic process which leads to TG increase during daily food intake is mainly mediated by the role of insulin, as this hormone mediates Apo48 synthesis as well as VLDL1 secretion from the liver [36]. It derives from the occurrence of abnormal triglyceride-rich lipoprotein when insulin resistance is inflamed [36].

Therefore, secondary conditions are mainly represented by the following: 1. the clinical condition/daily behavior able to increase TG levels (dietary intake of foods rich in TGs, alcohol abuse, metabolic syndrome, obesity, diabetes, pregnancy, hypothyroidism, kidney failure, Cushing syndrome, and/or acute hepatitis); 2. rare genetic causes (alteration in glycogen metabolism, autoimmune disease, Human Immunodeficiency Virus [HIV] infections); and 3. drug-induced HTG (hormone replacement therapies, beta-blockers, HIV-inhibitors, etc.) (Figure 2).

Indeed, the primary causes for HTG mainly derive from primary, genetic alterations in gene encoding for proteins involved in lipid metabolism. Fredrickson and Lees originally described five different pathological conditions which were characterized by the increase in plasma levels of specific lipoproteins and, therefore, their contents in TGs, CE, and/or phospholipids (P) [33].

Indeed, each alteration in genes involved in the metabolism of TG might dramatically impact the occurrence of HTG. A comprehensive knowledge about the genetic landscape of HTG is the mainstay for the development of pharmacological treatments able to dramatically impact the prognosis of these patients. Molecular target therapies are in phase II and III ongoing trials, whose interesting results will provide physicians with efficient weapons for counteracting HTG [38,39,40,41,42].

### 4.1. The Genetic Landscape of Familial Chylomicronemia Syndrome (FCS)

FCS is a rare inherited disease which is characterized by alterations in the metabolism of chylomicrons and their accumulation into the bloodstream [43]. FCS is mainly related to autosomal recessive disorder, characterized by homozygous or compound heterozygous mutations of the LPL gene which are responsible for the loss of function of the protein, thus leading to increased plasma concentrations in chylomicrons [44]. LPL is located on chromosome 8 and the occurrence of its mutations leading to FCS is around 1 every 100,000–1,000,000 individuals [44,45]. Indeed, FCS might occur in relation to the deficiency of the products of other further genes. An autosomal recessive inheritance pattern was related to FCS. Specifically, the loss of function of Apo-CII (gene APOC2, chromosome 19) and/or Apo-AV (gene APOA5, chromosome 11) promotes FCS, as these proteins are fundamental co-factors for the activation of LPL [44,45]. Deficiency in lipase maturation factor 1 (gene LMF1, chromosome 16)—i.e., a protein which is located in the ER and is responsible for the folding and assembly (i.e., maturation) of LPL [46]—might provoke impaired LPL function and increase plasma levels in triglyceride-rich lipoproteins. Similar results were derived from the deficiency in glycosylphosphatidylinositol-anchored high-density lipoprotein-binding protein 1 (gene GPIHBP1, chromosome 8) [44,45]. GPIHBP1 is a protein able to mediate the transfer of LPL from the interstitial spaces to the capillary lumen of the vessels [47]; a lack of function of this protein promotes severe HTG due to the entrapment of LPL into the interstitial spaces. 

**Figure 3:**
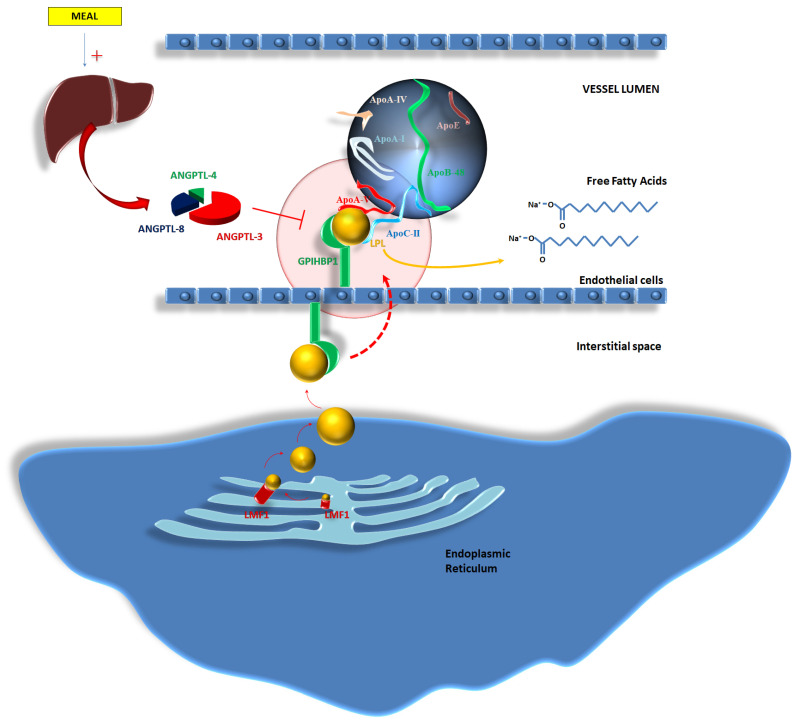
Lipoprotein lipase (LPL) complex: A reappraisal of the genetic background of LPL action. Abbreviations: ANGPTL-4,-8-3: Angiopoietin-Like Proteins -4,-8,-3; ApoA-I: apolipoprotein A-I; ApoA-IV: apolipoprotein A-IV; ApoA-V: apolipoprotein A-V; ApoB-48: apolipoprotein B-48; ApoC-II: apolipoprotein C-II; ApoE: apolipoprotein E; ApoE-II: apolipoprotein E-II; CM: chylomicron; GPI-HBP1: glycosylphosphatidylinositol-anchored high-density lipoprotein-binding protein 1; HIV: Human Immunodeficiency Virus; LFM1: Chaperone protein for maturation of LPL; LDL: low-density lipoprotein; LPL: lipoprotein lipase; RXR: retinoid X receptor; TG: triglycerides; VLDL: very low-density lipoprotein.

Furthermore, the activity of LPL is influenced by further enzymes which are a part of the lipasin/angiopoietin-like (ANGPTL) family. Specifically, feeding promotes an increased production in ANGPTL-3, -4 and -8 from the liver; ANGPTL-8 concentrations increase soon after a meal, leading to the activation of the ANGPTL-3 and -4 pathways. The complex mediates the inhibition of LPL activity [48]. Mutations able to inactivate ANGPTL-3 (chromosome 1p31.3 [49]), ANGPTL-4 (chromosome 19p13.2 [50]), and ANGPTL-8 (chromosome 19p13.2 [50]) reduce the TG plasma concentrations due to the enhancement of LPL activity [51,52,53]. Figure 3 tries to better explain the mechanisms related to the activity of LPL, the influence of co-factors on LPL activity, and the impact of the alterations in gene expression and protein transcription on the occurrence of FCS.

### 4.2. Familial Dysbetalipoproteinemia: Genetics and Molecular Mechanisms

The mutations in the apoE gene might influence the occurrence of Familial Dysbetalipoproteinemia (FDBL), i.e., a pathological condition characterized by fasting and postprandial HTG due to the reduced clearance of lipoprotein remnants [54,55].

The ApoE gene is located on chromosome 19q13.2 [55,56]. Three isoforms are derived: apoE2, apoE3, and apoE4, whose biosynthesis is under the control of three different alleles [55]. ApoE plays a key role in the clearance of remnants of chylomicrons and VLDL. Specifically, chylomicron remnants are cleared after interacting with LDLR, LRP, and heparan sulfate proteoglycans (HSPGs), but the interaction is mediated by apoE, which acts as a co-factor [56]. ApoE is further able to modulate the degradation of TGs via LPL [56].

Therefore, variations in apoE function and structure might lead to alterations in the metabolism of chylomicron remnants. The isoform ε2/ε2 of the apoE2 is mainly related to the recessively inherited form of FDBL [55,57], although the concurrence of hormonal disturbances, obesity, and/or age influence the phenotypic expression of this disease. Mutations in the ε2/ε2 APOE isoform might be observed in most of cases of FDBL, while in 10-15% of FDBL patients, these might derive from further variants in the gene sequence [57]. The phenotypic expression of FDBL is complicated by the low penetrance of the disease which avoids the clear expression of FDBL, even in the same familiar group [57]. 

### 4.3. Familial Hypertriglyceridemia and Combined Hyperlipidemia

Familial Hypertriglyceridemia (FHT) and Combined Hyperlipidemia (FCH) are two pathological entities characterized by moderately increased TG and LDL-C plasma levels [58]. It is hard to understand the exact genetic landscape of both of them, as the origin of these diseases might be considered as polygenic. Alterations in LPL, APOA5, APOC2, GPIHBP1, and LMF1 in FHT or APOA1-C3-A4-A5, APOE, USF1, TNFRSF1B, and LDLR in FCH had been considered as possible sources of the origin of these diseases, although the coexistence of secondary cardiovascular risk factors might trigger the phenotypic occurrence of cardiovascular complications [58].

Specifically, while FHT is mostly related to an increased production of VLDL particles from the hepatocytes, FCH is mainly characterized by the dramatic reduction in the clearance of remnants lipoproteins, which might promote atherosclerosis progression [59].

## 5. The Role of Hypertriglyceridemia in Cardiovascular Diseases

### 5.1. Triglycerides and Endothelial Function

The relationship between altered triglycerides metabolism and impairment in endothelial function is established [60,61], although the exact mechanisms are poorly understood [62].

Giannattasio et al. [63] noticed that high-fat meals in patients with untreated mild hypertriglyceridemia and dyslipidemia reduced the increase in radial artery diameter after 4-minute ischemia of the hand as compared to controls. As the increase in radial artery diameter is mainly related to nitric oxide (NO) release from endothelial cells due to the increased shear stress, authors thus supposed that high-fat meals might impair endothelial function in mild hypertriglyceridemic patients as compared to controls [63].

Indeed, the impairment in endothelial function in patients with hypertriglyceridemia might be related to further mechanisms. TGs are able to act on endothelium via p38 mitogen-activated protein kinases (p38MAPK) phosphorylation and activation [64]. Such a biochemical pathway is enhanced in the case of higher plasma levels of TGs which in turn promotes the activation of the nuclear factor kappa-light-chain-enhancer of activated B cells (NF-kB) and the cAMP response element-binding protein (CREB) [64]. CREB and NF-kB increase the transcription of genes deputed to the creation of proteins such as vascular cell adhesion molecule 1 (VCAM-1), endothelial leucocyte adhesion molecule-1 (ELAM-1), platelet endothelial cell adhesion molecule (PECAM-1), P-selectin, monocyte chemoattractant protein-1 (MCP-1/CCL2)—all implied in cell adhesion and migration into the intima layer—and ADAM metallopeptidase with thrombospondin type 1 motif (ADAMTS1), which is able to induce the proliferation/migration of vascular smooth muscle cells (VSMCs) and matrix degradation [64,65,66].

In parallel, TG-rich lipoprotein (TGRL) lipolysis is able to increase VCAM1 production via ER stress [67]. Specifically, Wang et al. [67] found increased ER stress after TGRL internalization and lipid droplet formation, which in turn promote the activation of the ER stress sensors PKR-like ER-regulated kinase (PERK) and inositol requiring protein 1α (IRE1α). PERK and IRE1α activation induced the expression of interferon regulatory factor 1 (IRF-1) which is a regulatory protein for the Vcam-1 gene [67].

The main actor in TGRL is surely the content in the cholesterol of the remnants and the specific characteristics of the lipoproteins [37]. Particles may enter the intima–media interface via migration through the endothelium as previously outlined. Generally, TGRL has a high content in cholesterol as compared to LDL [37]. The penetration of these particles might recruit monocyte–macrophages in order to clear this vascular section from TGRL. The net consequence is the creation of foam cells which enhance the atherosclerotic process and the vascular inflammatory pathways at the origin of the plaques [37]. Bermudez et al. [68] identified an upregulation in apoB48 receptor mRNA synthesis in macrophages soon after the increase in TGRL content, which in turn promotes the enhanced phagocytosis of these lipoproteins by macrophages themselves. Furthermore, TGs seem to promote the production of IL-1β, IL-6 and PGE2, and the phagocytic capacity of macrophages, thus impacting their transformation in foam cells [69]. Finally, recent research evaluated the role of the VLDL receptor on macrophages in the promotion of transformation in foam cells, although dedicated clinical studies should aim to better demonstrate it [70].

The impact of increased TGRL lipolysis on endothelial function is a further mechanism for understanding the role of TG on vascular walls. Eiselein et al. [71] observed the decrease in endothelial barrier function after TGRL lipolysis: the distribution of zonula occludens-1 (ZO-1) proteins was altered, as well as F-actin. Meanwhile, TGRL might promote the increase in caspase-3 activity, which in turn might provoke nuclear fragmentation and thus endothelial apoptosis [71]. Shin et al. [72] demonstrated endothelial apoptosis in human umbilical vein endothelial cells (HUVECs) which were exposed to TGRL. Specifically, the activation of a lectin-like oxidized low-density lipoprotein receptor-1 (LOX-1) seemed to mediate the increased production of tumor necrosis factor (TNF)-α and interleukin-1β, DNA fragmentation, and thus cell death [72].

The release of fatty acids and the nature of fatty acids seem able to influence endothelial function. Halle et al. [73] found that palmitic acid and oleic acid reduced the activity of endothelial nitric oxide synthase (eNOS), while linoleic acid did not impact on eNOS function. Therefore, hypertriglyceridemia and its derivative products differently alter vascular function in relation to the type of the free fatty acid coming from lipoprotein release [73].

Wang et al. [74] observed that the lipolysis of TGRL might first alter the morphology of the endothelial surface by altering the distribution of low-density lipoprotein receptor-related proteins, endothelial nitric oxide synthase, and caveolin-1. Secondly, TGRL lipolysis was associated with increased reactive oxygen species (ROS) production due to the enhanced activation of the nicotinamide adenine dinucleotide phosphate (NADPH) enzyme [74].

Rosenblat et al. [75] effectively demonstrated increased ROS production after macrophage triglyceride accumulation which, on the contrary, promoted the upregulation of the expression of the Human Paraoxonase-2 (PON2). PON2 shows antioxidant skills as it reduces oxidative stress in mitochondria and in the endoplasmic reticulum, and further inhibits apoptosis and prevents the formation of atherosclerotic lesions [76]. Nevertheless, PON2 upregulation through macrophage triglyceride accumulation seemed to be mediated by the activation of janus kinase (JNK) but not extracellular signal-regulated kinase (ERK), which resulted in c-Jun phosphorylation when higher ROS concentrations were in the vessels layers [75].

### 5.2. Triglycerides and Cardiac Muscular Cells

The impact of TGs on cardiac muscular cells and, consequentially, on cardiac function and morphology, is still a matter of debate [77]. Preclinical data suggested the negative impact of TG on the healthy performance of cardiac muscular cells in their daily clinical activities.

In vitro models involving human cardiomyocyte-derived cells which were exposed to different fatty acids (FA) from TGs of the diet demonstrated the impact of FAs on ER stress [78]. Specifically, palmitate seemed to upregulate the expression of genes such as the DNA damage-inducible transcript 3, also known as C/EBP homologous protein (CHOP), the activating transcription factor 6 (ATF6), and Glucose-regulated protein (GRP78), as well as the phosphorylation of eukaryotic initiation factor 2a (EIF2a); these modifications in the gene expression of cardiac muscular cells turn into increased ER stress, which might favor the occurrence of irreversible cell damages and apoptosis [78,79].

Although TGs might serve cardiac cells in sustaining energy supply via β-oxidation processes, excess in TGs plasma levels and inclusion in muscular cells turn into lipotoxic effects [80]. The expression of MTTP influences these effects. MTTP is involved in the assembly of apoB-containing triglyceride-rich lipoproteins [81]. The increase in MTTP provokes the transferal of TGs in apoB-lipoproteins, thus reducing the burden of accumulation of TGs into cardiac cells, without impacting on insulin sensitivity but rather reducing the risk of lipotoxic damages to them [82].

Indeed, alterations in TGs metabolism in cardiac muscular cells are multifactorial. Hypertensive status might influence TGs metabolism in heart cells by promoting a lowering expression of proteins such as adenosine monophosphate-activated protein kinase (AMPK) phosphorylation and peroxisome proliferator-activated receptor-(PPAR)α protein content, thus indicating the reduced activity of the β-oxidation pathway [83]. Furthermore, hypertension induced a decreased protein content of α/β-hydrolase domain-containing 5, an adipose triglyceride lipase (ATGL) activator, and an increased content of G0/G1 switch protein 2, an ATGL inhibitor, which suggests the reduced activity of lipolytic pathways in the heart of hypertensive individuals [83].

The imbalance in ATP synthesis might explain the possible reduction in contractile function as seen in the hearts of mice with TG-rich cardiac cells [84].

These results might be partially explained by alterations in the expression and function of LPL on the surface of cardiomyocytes. Yagyu et al. [85] created transgenic mice which were able to produce altered lipoprotein lipase on the surface of cardiomyocytes. The results first reported an accumulation of lipids in cardiac muscular cells and, second, alterations in the morphology of the myocytes, which were enlarged and unable to provide an efficacious contractile function, thus inducing cardiac ventricle dysfunction [85].

Kankaanpää et al. [86] effectively reported that FFA exposure was mainly related to left ventricular mass while demonstrating a negative correlation with the cardiac index and a positive correlation with peripheral vascular resistance. Although Szczepaniak et al. [87] found higher cardiac TG concentrations in obese patients, the exact role of TG in promoting alterations in cardiac function is still under investigation. A study [80] revealed reduced concentrations in TGs in failing myocardial cells, while increased concentrations in ceramides were further observed. It was supposed that the inhibition in Akt-signaling and abnormalities in AMPK biochemical pathway might provoke increased concentrations in ceramides in cardiac cells, thus predisposing to failure [88]. Alterations in Kruppel-like factor 15 have been considered as possible physiopathologic mechanisms in the impairment of cardiac cells in patients with lipid dysregulation [89]: dysfunctional Kruppel-like factor 15 promoted impaired lipid utilization, the excessive activity of prohypertrophic proteins, and pathologic hypertrophy with stress [89], while Kruppel-like factor 4 influenced the transcriptional control of cardiac mitochondrial homeostasis [90]. Therefore, increased TGs and the altered cardiac influx of them into cardiac cells due to Kruppel-like factors might provoke failure in cardiac muscular cells. 

Perilipin 2 (Plin2) might influence TGs accumulation in cardiac cells. Plin2 is a lipid droplet protein which mediates the creation and movement of lipid droplets and enforces stability [91]. A higher expression in Plin2 is related to cardiac steatosis, but Plin2−/− mice showed an increased accumulation in TGs in cardiac cells and an exacerbated reduction in stroke volume and cardiac output after cardiac ischemic injury, as compared to Plin2+/+ mice [91]. This condition seemed mediated by altered lipophagy, which impaired the degradation of lipid droplets, thus resulting in cardiac steatosis.

Indeed, the accumulation of TGs in cardiac cells might also be related to alterations in the cardiomyocyte circadian clock [92]. Specifically, Tsai et al. [92] observed the inactivation of LPL—probably mediated by AMPK activation—and an increase in TGs synthesis during and at the end of the active/awake phase, respectively. The consequence was a marked increase in cardiac myocytes volume and an induction of cardiac steatosis. 

## 6. Conclusions

Hypertriglyceridemia still represents a neglected cardiovascular risk factor which is related to worse outcomes and major adverse cardiac events. Higher plasma concentrations in TGs might alter endothelial function and promote cardiac steatosis, myocardial impairment, and—definitely—irreversible damages to heart. The exact mechanisms which might explain why such modifications are poorly understood. Further research is needed in order to understand the molecular basis of cardiac and vascular damages from hypertriglyceridemia to promote the identification of specific compounds able to efficaciously counteract the progress of lesions in these patients.

## Data Availability

Not applicable.
